# Implementation of a vascular access specialist team in a tertiary hospital: a cost-benefit analysis

**DOI:** 10.1186/s12962-023-00464-6

**Published:** 2023-09-16

**Authors:** Laura Ricou Ríos, Candela Esposito Català, Arnau Pons Calsapeu, Cristina Adroher Mas, Isabel Andrés Martínez, Isaac Nuño Ruiz, Mònica Castellà Creus, Laia Castellà Fàbregas, Maria José García Quesada, Oriol Estrada Cuxart, Jordi Ara del Rey, Francesc López Seguí

**Affiliations:** 1 Direcció d’Estratègia Assistencial, Gerència Territorial Metropolitana Nord, Institut Català de la Salut, Barcelona, Spain; 2 CRES - Centre de Recerca en Economia de la Salut, Universitat Pompeu Fabra, Barcelona, Spain; 3 Research Group on Innovation, Health Economics and Digital Transformation, Institut Germans Trias i Pujol, Barcelona, Spain; 4 Hospital Universitari Germans Trias i Pujol, Institut Català de la Salut, Barcelona, Spain; 5 Direcció Infermera. Àrea de Qualitat. Gerència Metropolitana Nord, Hospital Germans Trias i Pujol, Institut Català de la Salut, Barcelona, Spain; 6 NURECARE-IGTP Nursing Research Group, Germans Trias i Pujol Research Institute, Badalona, Spain; 7 Gerència Territorial Metropolitana Nord, Institut Català de la Salut, Barcelona, Spain

**Keywords:** Catheters, Cost benefit analysis, Economic evaluation, Healthcare-associated infections, Nursing care, Peripherally inserted Central Catheter, Peripheral venous catheters, Ultrasound

## Abstract

**Background:**

The use of peripherally inserted central catheters and midline catheters is growing due to their potential benefits. These devices can increase patient safety and satisfaction while reducing the use of resources. As a result, many hospitals are establishing vascular access specialist teams staffed by nurses who are trained in the insertion and maintenance of these catheters. The objective of the study is to evaluate previously to the implementation whether the benefits of introducing ultrasound-guided peripheral venous catheters, midline catheters and peripherally inserted central catheters compared to current practice by a vascular access specialist team outweigh their costs.

**Methods:**

Cost-benefit analysis from the perspective of the healthcare provider based on administrative data. The study estimates the reduction in resources used when changing the current practice for the use of ultrasound-guided midline and PICC catheters, as well as the additional resources required for their use.

**Results:**

The use of an ultrasound-guided device on peripherally inserted central carheter, results in a measurable resource reduction of approximately €31. When 3 peripheral venous catheters are replaced by an ultrasound-guided peripherally inserted central catheter, the saving is €63. Similarly, the use of an ultrasound-guided device on a midline catheter, results in a reduction of €16, while each ultrasound-guided midline catheter replacing 3 peripheral venous catheters results in a reduction of €96.

**Conclusion:**

The benefits of using ultrasound-guided midline and PICC catheters compared to current practice by introducing a vascular access specialist team trained in the implantation of ultrasound-guided catheters, outweigh its cost mainly because of the decrease in hospital stay due to the lowered risk of phebitis. These results motivate the implementation of the service, adding to previous experience suggesting that it is also preferable from the point of view of patient safety and satisfaction.

## Background

Peripheral venous catheters (PVC) are the most widely used invasive devices in hospitals, with a prevalence rate of 66% among patients admitted to Catalan hospitals [1, 2]. However, they are often used excessively and inappropriately, particularly when the peripheral intravenous line is not the preferred treatment option [3]. Furthermore, PVCs are associated with a high incidence of minor local complications, resulting in additional costs for both patients and healthcare providers [4]. Research indicates that over 73% of patients may contract a healthcare-associated infection (HAI) due to the presence of a PVC [5]. Additionally, some studies suggest that retaining an unused catheter in a patient may increase the risk of developing potentially avoidable complications by more than 25% [6]. The most relevant complications that require catheter replacement include phlebitis, obstruction, infiltration and extravasation [4, 7].

On the other hand, the use of peripherally inserted central catheters (PICC) and midline catheters is growing due to the numerous benefits they offer to patients and healthcare providers. In the first case, they increase patient safety and satisfaction by preserving venous capital and eliminating the discomfort caused by multiple venous punctures [8, 9]. In the second case, they could optimize the use of resources if a PICC or a midline catheter could replace various PVC.

Due to the numerous benefits of using PICCs and midline catheters, as well as the drawbacks associated with PVC, more hospitals are creating vascular access specialist teams (VAST). These teams are formed by nurses who are specially trained in the insertion and maintenance of PICCs and midline catheters, utilizing ultrasound support during insertion [10]. Several studies suggest that this method is more cost-effective [11, 12]: on one hand, it offers greater accuracy during insertion and a higher success rate of venipunctures, which eliminates the need of an X-ray to confirm correct catheter placement [13]; on the other hand, patients are less likely to develop HAIs [5]. In addition, it increases patient satisfaction due to greater comfort, reduced pain sensation, greater patient mobility and decreased length of hospital stay [11, 13, 14]. Nevertheless, the Cochrane Library conducted a systematic review to compare the effectiveness of the vascular access specialist team with the generalist model commonly used in hospitals and other medical centers, concluding that there is currently insufficient high-quality evidence to either support or reject the implementation of a VAST [15].

Healthcare-associated infections affect 5–10% of patients admitted to a hospital [13] and are a major burden on the healthcare system, as when they occur they lead to a 77% increase in cost per patient (31% of this increase caused by pharmacy costs, 31% by materials and services, 24% by increased hospital stay and 14% by laboratory costs) [16]. In addition, HAIs are associated with additional complications, which in the worst-case scenario can result in the death of the patient. Some studies estimate that 6% of patients with HAIs eventually die from it [17]. The most common HAIs associated with catheter use are bacteremia and phlebitis of infectious origin [13]. In the case of bacteremia, an episode due to a PVC infection, might increase hospital costs by €18,078 [18] and the patient’s stay tends to be longer and much more expensive [19]. Moreover, if it happens, the probability of mortality rises to 1.7% [20, 21]. In case of infectious phlebitis due to PVCs, several studies report a prevalence between 3% and 12% [3, 22].

Despite the economic impact of HAIs on the healthcare system, there is a lack of up-to-date data in Catalonia that can accurately determine the direct and indirect costs of this type of infections. In 2015, it was estimated that the cost represented by PVC bacteremia in Catalan hospitals was above 12 million euros, a cost that had already been reduced by approximately 10 million euros during the 2008–2013 period thanks to the implementation of healthcare-associated infection surveillance initiatives such as the VINCat Programme, the Bacteremia Zero Programme and the local programmes initiated by care teams [23].

**Fig. 1:**
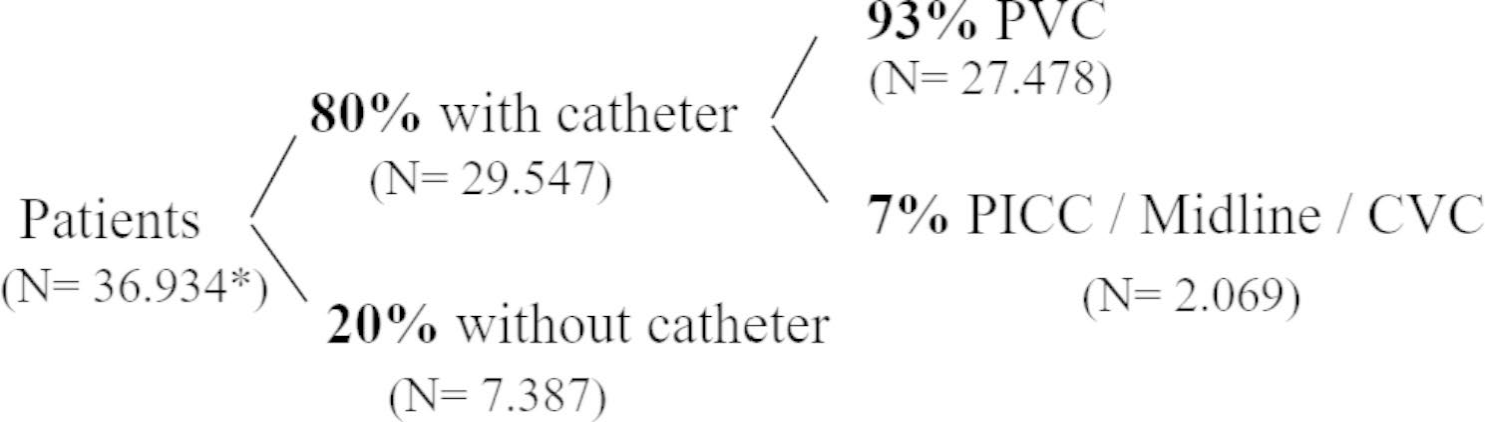
Number of patients with catheters annually in the Germans Trias i Pujol University Hospital. * Number of patients discharged in 2019

The Germans Trias i Pujol University Hospital is a public healthcare center that provides highly complex medical care to a reference population of 800,000 inhabitants in the Northern Metropolitan Health Region of Barcelona. The hospital is part of the Catalan Health Institute, which is the largest provider of healthcare services in Catalonia. In 2022, despite the widespread use of venous catheters (approximately 80% of its patients carry one at some point during their hospital stay), the hospital has not yet implemented a VAST. The most commonly used catheters were PVCs (93%), while other types like PICCs, midline catheters and CVCs were used less frequently (Fig. 1). A survey conducted in 2019 by the hospital’s Infection Control Unit indicated that 71% of nurses do not use PICCs and midline catheters when they are indicated, despite their potential benefits to patients and the hospital. Nurses cited a lack of skills and the pressure of care as the main reasons for not using these devices.

In this context, given the potential benefits of using an ultrasound-guided technique for the insertion of PICCs and midline catheters, the hospital is considering adopting a VAST. The aim of this study is to conduct a cost-benefit analysis of the use of ultrasound-guided PVC, midline and PICCs compared to current practice, in order to demonstrate the important economic impact of this measure before implementing it.

## Methods

### Description of the intervention

The team will attend patients who are hospitalized in the semi-critical and acute care units, as well as those receiving care at home or in the emergency room. Patients admitted to critical care units will not be attended by the team, as the nursing professionals in those units are already experienced in ultrasound-guided insertion of this type of catheter. Moreover, as most of the CVCs are only used in this unit, the new team will not replace this type of catheter. The decision regarding the most suitable type of catheter for a patient is determined by a hospital-implemented decision algorithm. Factors such as medication pH and osmolarity levels, potential damage to the vein’s intimal layer, type and duration of treatment and the patient’s clinical history are taken into consideration by the algorithm to determine the appropriate type of catheter and the need for team intervention. In addition, The team will work in collaboration with the Healthcare-associated infections Infection Control Nursing Team to develop and evaluate protocols related to the correct use of the different types of catheter and will monitor the evolution of clinical evidence and implement changes in practice accordingly.

The team will comprise two nursing professionals who will be replaced in case of illness, incapacity or vacation, following the model of other hospitals with a VAST. They will not work night or weekend shifts. If the algorithm indicates the team’s intervention is required for patients hospitalized during these time slots, an alternative catheter will be placed until the team returns.

Based on estimations provided by the hospital’s nursing staff, each team member can insert an average of five catheters per day, considering insertion time and experiencies from other hospitals. With two team members working 250 days a year, the hospital’s annual capacity for catheter insertions would be 2,500. Figure 1 indicates a higher number of patients requiring catheters, but the algorithm will determine the exact number to be inserted by the team. Increasing the number of team members will depend on their performance and capacity to attend all patients recommended by the algorithm.

### Study design

A cost-benefit analysis was conducted from the healthcare provider perspective, previously to the implementation of the team. On one hand, it has been estimated the reduction in resources used when changing the current practice for the use of ultrasound-guided devices. On the other hand, the additional resources required for the use of the ultrasound-guided PICCs and midline catheters have also been studied. The analysis followed the Consolidated Health Economic Evaluation Reporting Standards [24].

### Data

The total costs of the ultrasound-guided PICCs and midline catheters placement were calculated by quantifying the material costs and the practitioner’s time burden associated with catheter placement, assuming a PICC or a midline catheter could replace an average of three PVCs. In contrast, the total benefits were calculated by quantifying the reduction in resources used from eliminating confirmatory radiography and PVCs material, as well as the reduction in staff burden. Additionally, the reduction of bacteremia and hospital stay due to the elimination of phlebitis was also quantified. The costs were obtained from the hospital administrative database and were measured in monetary units (euros 2021). No discount rate was used.

### Outcome measures

Benefits and costs have been classified into the following categories: personnel, material, confirmatory radiographies and possible complications (Table 1).

**Table 1:** Categories of costs

Cost category		Cost (EUR)	Unit
**Personnel**	Vascular Access Specialist Team	€ 53,583	Annual cost to company per employee
	Personnel burden PICC	€ 32	Per procedure
	Personnel burden PICC with ultrasound-guided technique	€ 21	Per procedure
	Personnel burden midline	€ 21	Per procedure
	Personnel burden midline with ultrasound-guided technique	€ 10	Per procedure
	Personnel burden PVC	€ 12	Per procedure
**Material**	Material price PICC	€ 56	Per procedure
	Material price midline	€ 34	Per procedure
	Material price PVC	€ 2	For 3 procedures
**Radiography**	Confirmatory radiography PICC	€ 15	Per procedure
**Possible complications**	Increased length of stay due to phlebitis	€ 674	Per day of hospital stay
	Increased resource use due to bacteremia	€ 18,078	Per bacteremia

In terms of personnel costs, the cost of hiring each nursing professional for the VAST is estimated at approximately €53,583 (annual cost to the company). The monetary value of workload relief for the nursing staff provided by the new team was calculated by multiplying company’s hourly cost supported for each worker (€32.63 per hour) by the time required for professionals to perform the insertion of different catheters: approximately 60 min for PICC, 40 min for ultrasound-guided PICC, 40 min for a midline catheter, 20 min for an ultrasound-guided midline catheter and 22.5 min (7.5 min multiplied by an average of 3 peripheral venous catheters insertions per episode) in the case of PVC. The result of the quantification of the workload relief is €32.63 per intervention for a PICC, €21.75 per intervention for an ultrasound-guided PICC, €21.75 per intervention for a midline catheter, €10.88 per intervention for an ultrasound-guided midline catheter and €12.24 per 3 PVCs insertions.

The cost of PICC material is close to €56.31, which is calculated as the weighted average of the prices of different types of PICCs used in the hospital in 2021. The cost of materials for an ultrasound-guided PICC is the same. Similarly, the material cost for a midline catheter is approximately €34, which is the same as the material cost for an ultrasound-guided midline catheter. The cost of the PVC material is approximately €0.68 (€2.06 for three units). The cost of the ultrasound scanner used for ultrasound-guided insertion is not included, as it is accounted for in the cost of the material used for each catheter placement. The cost of the X-ray required to verify the correct placement of the PICC is estimated to be around €15 [25].

Finally, it has been taken into account the potential benefits resulting from the reduction of complications and infections through the incorporation of trained professionals for the introduction of ultrasound-guided PICCs and midline catheters. Firstly, replacing PVCs with PICCs or midline catheters may lead to a reduction in the length of hospital stay due to decreased infections and complications. The value of this reduction in length of stay was estimated by multiplying the probability of phlebitis occurring with PVCs (12% according to our own data) with the average cost of increased length of stay (€674 per 24-hour stay [25], for an estimated increase of 1.5 days, based on our own data). Secondly, the decrease in the number of bacteremias associated with both PVCs and PICCs/midline catheters implies a reduction in resource utilization. This value was calculated by multiplying the number of episodes of bacteremias related to the selection and insertion of PVCs and PICCs/midline catheters at the Germans Trias i Pujol University Hospital (9 in 2021, according to our own data) with the cost associated with a patient developing this infection (€18,078) [18], and then dividing this value by the total number of patients with any type of catheter (29,547). Therefore, the reduction in the number of bacteremias derived from replacing every three PVCs or one PICC with an ultrasound-guided PICC, or every three PVCs or one midline catheter with an ultrasound-guided midline catheter, represents a quantifiable reduction in resources of approximately €5.50.

## Results

Table 2 shows the benefits and costs of introducing an ultrasound-guided device on PICC and midline catheters, as well as the ones derived from replacing PVCs with ultrasound-guided PICCs and midlines. We observe that the use of an ultrasound-guided device on peripherally inserted central carheter, results in a measurable resource reduction of approximately €31. When 3 peripheral venous catheters are replaced by an ultrasound-guided peripherally inserted central catheter, the saving is €63. Similarly, the use of an ultrasound-guided device on a midline catheter, results in a reduction of €16, while each ultrasound-guided midline catheter replacing 3 peripheral venous catheters results in a reduction of €96.

The replacement of three PVCs, either with a PICC or a midline catheter, results in the most significant reduction in resources, especially in the second case. Moreover, as can be seen in the last column of Table 2, in both cases the major driver for this reduction is the potential decrease in hospital stay due to the lowered risk of phlebitis, which accounts for 86% of the benefits. This is primarily because the cost per day of hospitalization is substantial. On the other hand, although the cost of a patient with bacteremia is the highest, its magnitude is not reflected in the results, as the probability of its occurrence is relatively low.

**Table 2:** Benefits and costs of substitution

		Benefit/Cost of substitution	% of reduction
**Substitution of PICC with ultrasound-guided PICC**	Reduction of personnel burden	€ 32	62%
	Reduction of bacteremia	€ 5	10%
	Confirmation X-ray elimination	€ 15	29%
	**Reduction of resources**	**€ 52**	**100%**
	Increase of personnel burden	€ -21	
	**Increase in resources**	**€ -21**	
	**Total**	**€ 31**	
**Substitution of 3 PVC with 1 ultrasound-guided PICC**	Reduction of personnel burden	€ 12	9%
	Reduction of bacteremia	€ 5	4%
	Elimination of PVC material	€ 2	1%
	Reduced hospital stay due to elimination of phlebitis	€ 121	86%
	**Reduction of resources**	**€ 140**	**100%**
	Cost of PICC	€ -56	
	Increase of personnel burden	€ -21	
	**Increase in resources**	**€ -77**	
	**Total**	**€ 63**	
**Substitution of midline with ultrasound-guided midline**	Reduction of personnel burden	€ 21	81%
	Reduction of bacteremia	€ 5	19%
	**Reduction of resources**	**€ 26**	**100%**
	Increase of personnel burden	€ -10	
	**Increase in resources**	**€ -10**	
	**Total**	**€ 16**	
**Substitution of 3 PVC with 1 ultrasound-guided midline**	Reduction of personnel burden	€ 12	9%
	Reduction of bacteremia	€ 5	4%
	Elimination of PVC material	€ 2	1%
	Reduced hospital stay due to elimination of phlebitis	€ 121	86%
	**Reduction of resources**	**€ 140**	**100%**
	Cost of midline	€ -34	
	Increase of personnel burden	€ -10	
	**Increase in resources**	**€ -44**	
	**Total midline**	**€ 96**	

## Discussion

The economic analysis of implementing a vascular access specialist team at the Germans Trias i Pujol University Hospital suggests that it can result in significant resource savings when using ultrasound-guided catheters compared to current practice, while concurrently improving patient safety and well-being by reducing the risk of phlebitis and bacteremia. The replacement of PVCs with PICCs or midline catheters yields the highest reduction in resources, especially in the second case. Moreover, in both cases, the primary driver of cost reduction is the potential decrease in hospital stay due to lower risk of phlebitis. These findings suggest that prioritizing the replacement of PVCs, rather than PICCs or midline catheters inserted without ultrasound guidance, is more beneficial for both patients and the hospital.

Previous studies have concluded that the use of ultrasound-guided PICCs is cost-effective [8–10]. However, to our knowledge, there is no evidence of studies analyzing the cost-benefit of the implantation of a VAST. Gosselin et al. [26] analyzed the cost-effectiveness of the introduction of a VAST specialized in the insertion of ultrasound-guided PICCs, and found that the reduction in resources derived from using the device was approximately double from our study (€65 vs. €31). Although this reduction is similar in terms of material resources, in contrast to our study, they considered the cost of disinfecting the room and patient transport, but did not incorporate the increase in resources derived from HAIs. Tan et al. [11] have also analyzed the cost-effectiveness of ultrasound-guided PICCs. They suggested that the benefits become visible six months after introducing the team in the hospital. They also highlighted the greater comfort for patients and the reduced time for catheter insertion and complications associated with the new catheters. In this sense, a study carried out in Catalonia has estimated the cost of a patient with bacteremia, suggesting an increase in resources of €18,078 [18], although other studies indicate a potentially higher cost of €21,506 [27]. These findings suggest that the cost savings associated with introducing the team in our study may be conservative, and the actual cost benefits could potentially be even greater.

In the case of midline catheters, a study comparing their effectiveness with that of PVCs demonstrates that midline catheters significantly reduce complications associated with PVCs, such as phlebitis, infiltrations, asymptomatic thrombosis, occlusions and accidental removals. The study supports our conclusion and suggests that although midline catheters may have higher costs, these are compensated by the complications prevented, and that patients also prefer midline catheters [28]. A study conducted by Moulin et al. [29] proposes that ultrasound-guided midline catheters are a good alternative to PVCs in cases of prolonged treatment when PICCs placement is not feasible. Moreover, the study adds that, in addition to the reduction in the risk of HAIs, the main benefits for patients with this type of catheter are that it can be inserted at the patient’s bedside, does not require a confirmatory X-ray and allows to obtain repeated blood samples without additional punctures.

The hospital’s guidelines are in line with the recommendations of the Catalan Agency for Health Quality and Evaluation [1], which emphasize the appropriate use of catheters in clinical practice to reduce HAIs and improve patient satisfaction. According to this study, implementing a VAST could be a positive step towards achieving these goals. Future studies should estimate the impact of this change in the venous access device usage model on clinical variables and user satisfaction, as well as determine the number of catheters of each type that can be inserted by the team based on its size and decision algorithm. With this data, it will be possible to further evaluate the effectiveness of the introduction of the team, including whether the benefits achieved from using ultrasound-guided catheters outweighs the cost of hiring the team’s professionals.

### Limitations

This study has several limitations. First, some of the impacts of implementing a VAST are not easily quantifiable and are not accounted for in the calculation. In the case of PVCs replacement, these are reflected in the improvement in patient experience and well-being due to the easier catheter insertion, which preserves venous access and increases safety [14, 30]. In the case of PICCs and midline catheters substitution, the time to start the treatment is reduced and communication between the nurse and the patient is improved. Previous studies suggested that 83% of patients with the ultrasound catheters rate the change positively and 63% of patients highlight increased comfort with PICCs [31].

Secondly, in the absence of specific costs for the device placement procedure, it was assumed to be the same for PVCs, PICCs and midline catheters. Additionally, the costs of the supplies used for insertion, maintenance and replacement of catheters, were not considered, assuming that they could be similar between the compared models. Moreover, the cost of the catheter tip verification system was also not taken into account. Furthermore, the introduction of a VAST requires a cultural change and recognition of its activity by all professionals in the institution. Future studies could also incorporate costs associated with materials, communication and dissemination of corresponding information sessions.

Thirdly, it has been assumed that the productive capacity of the team corresponds to a 3:1 ratio in the PVC replacement and that the time required for catheter insertion in the hospital is comparable to the evidence shown by other hospitals. Future research should assess the reliability of these assumptions. Moreover, future studies should also evaluate the impact on the risk of HAIs of the introduction of the team.

## Conclusion

The economic impact of using ultrasound-guided midline and PICC catheters compared to current practice resulting from the implementation of a VAST has been analyzed and quantified, concluding that the monetary benefits outweigh the costs. Furthermore, the results suggest that prioritizing the substitution of PVCs with an ultrasound-guided midline or PICC, is more beneficial for both the patient and the hospital, mainly because the decrease in hospital stay due to the lowered risk of pheblitis.

This study is the first to quantify the economic impact of using ultrasound-guided devices resulting from the implementation of a specialized team in our setting. It provides an estimation of the costs and benefits of implementing such a team, while also establishing a conceptual and reference framework for evaluating the post-implementation results.

## Data Availability

All the data utilized in the study is explicitly documented within the text, without any supplementary data or dataset used to get the data documented in the text.

## Abbreviations

PVC: Peripheral venous catheters
HAI: Healthcare-associated infection
PICC: Peripherally inserted central catheters
VAST: Vascular access specialist teams
CVC: Central venous catheters
